# Five new *Camillea* (Xylariales) species described from French Guiana

**DOI:** 10.1186/s40529-023-00397-6

**Published:** 2023-10-28

**Authors:** Jacques Fournier, Huei-Mei Hsieh, Christian Lechat, Yu-Ming Ju, Delphine Chaduli, Anne Favel

**Affiliations:** 1 Las Muros, Rimont, 09420 France; 2 Institute of Plant and Microbial Biology, Academia Sinica, Nankang, Taipei, 115 Taiwan; 3 64 route de Chizé, 79360 Villiers-en-Bois, France; 4 INRAE, Aix-Marseille Université, UMR1163, 13288 Marseille, France

**Keywords:** Ascomycota, Graphostromataceae, Neotropics, Phylogeny, Saül, SEM, Taxonomy

## Abstract

**Background:**

The genus *Camillea* was created in 1849 from collections made in French Guiana with eight species included. Numerous species assigned to *Camillea* were subsequently discovered, especially in the forests of the Amazon basin, but new discoveries have not been reported from French Guiana since 1849. Recent fieldwork in French Guiana has begun to fill this gap by identifying five new species, most of which were collected in the vicinity of Saül village.

**Results:**

Based on macro- and micromorphological study of their stromata, including SEM images of ascospore wall ornamentation, five new species were recognized, including *C. cribellum, C. heterostomoides, C. nitida, C. rogersii* and *C. saulensis.* Cultures could be obtained for *C. heterostomoides* and *C. rogersii*, and ITS and LSU sequences were obtained for all of the five new species. *Camillea heterostoma* and its variety *microspora* were shown to be conspecific. Provisional molecular phylogenetic analyses support the possible reinstatement of *Hypoxylon melanaspis*, currently regarded as merely an applanate form of *C. leprieurii*.

**Conclusion:**

The current study is based on a relatively limited fieldwork in its duration and sampling area but was able to substantially increase the number of *Camillea* species known from French Guiana. This augurs an exceptional and still unknown diversity of the genus in this area and by extension in the adjacent neotropical forests.

## Background

In the mid-nineteenth century, French Guiana became the cradle of the new genus *Camillea* Fr., following the collections of eight different, remarkable and undescribed pyrenomycetous ascomycetes made by François Mathias René Leprieur in the vicinity of Cayenne during the years 1837–1839. The hectic nomenclatural history of *Camillea* is briefly summarized here. For a more complete overview, the reader is referred to Læssøe et al. (1989).

The genus *Camillea* was established by Fries (1849) to accommodate the material collected in French Guiana and first described by Montagne (1840) in *Hypoxylon* “tribe” (subgenus) *Bacillaria* based on their often more or less upright stromata. Since the broad concept first followed by Fries and the subsequent taxonomists, the delimitation of the genus has been gradually narrowed down by Dennis (1957), Pouzar (1979) and Læssøe et al. (1989) who respectively excluded species currently placed in *Phylacia* Lév., *Biscogniauxia* Kuntze and *Leprieuria* Læssøe, J. D. Rogers & Whalley. In addition, Pouzar (1979) included in *Camillea* species with applanate stromata and light colored ascospores previously accommodated in *Nummularia* Tul. & C. Tul. by some authors and in *Hypoxylon* section *Applanata* by Miller (1961). A recent phylogenetic revision of Xylariaceae by Wendt et al. (2018) resulted in the segregation from the Xylariaceae of the two distinct families Graphostromataceae M.E. Barr, J. D. Rogers & Y.-M. Ju. emend M. Stadler, L. Wendt & Sir and Hypoxylaceae DC. *Camillea* is currently placed in the Graphostromataceae along with *Biscogniauxia*, *Graphostroma* Piroz., *Obolarina* Pouzar and *Vivantia* J. D. Rogers, Y.-M. Ju & Cand.

*Camillea* was monographed by Læssøe et al. (1989) who defined it by the unique combination of the following features: (i) highly carbonaceous stromata erumpent through bark covered by a fleeting dehiscent ectostromatic layer of mixed bark and fungal tissues; (ii) asci with a usually massive rhomboid and amyloid apical apparatus; (iii) subhyaline to yellowish ascospores featuring a variously ornamented surface when observed by SEM, and (iv) a *Xylocladium*-like asexual morph.

Among the 65 names of *Camillea* listed in MycoBank, the removal of synonyms and excluded taxa results in 40 currently accepted taxa including two varieties. Eight of them were originally described by Montagne (1840, 1855) from French Guiana, and the remaining 32 taxa were subsequently introduced, their holotypes mostly from Central and South America (25) but none from French Guiana, and others from Malaysia (2), Thailand (1) and USA (4) (Table 1).

**Table 1:** Synoptic table of *Camillea* taxa where they were originally described with the taxon number in parentheses. Countries are arranged in descending order of the taxa number. Taxon names are followed by references where they were associated with *Camillea* for the first time

Country	Taxon names
French Guiana (8)	*C. cyclisca* (Læssøe et al. 1989), C. *cyclops* (Montagne 1855), *C. fossulata* (Læssøe et al. 1989), C. *heterostoma* (Læssøe et al. 1989), C. *labellum* (Montagne 1855), *C. leprieurii* (Montagne 1855), *C. mucronata* (Montagne 1855), *C. scriblita* (Læssøe et al. 1989)
Ecuador (5)	*C. amazonica* (Læssøe et al. 1989), C. *fusiformis* (Whalley 1995), *C. ovalispora* (Hastrup and Læssøe 2009), *C. unistoma* (Hastrup and Læssøe 2009), *C. verruculospora* (Rogers et al. 1991)
Costa Rica (4)	*C. coroniformis* (Rogers et al. 2002), C. *heterostoma* var. *microspora* (Rogers et al. 2002), C. *labiatirima* (Rogers et al. 2002), C. *obularia* (Rogers et al. 1991)
Mexico (4)	*C. guzmanii* (San Martín and Rogers 1993), *C. hyalospora* (Rogers et al. 1991), C. *magnifica* (San Martín and Rogers 1993), *C. mexicana* (San Martín and Rogers 1993)
Peru (4)	*C. deceptiva* (Læssøe et al. 1989), C. *oligoporus* (Læssøe et al. 1989), C. *patouillardii* (Læssøe et al. 1989), C. *stellata* (Læssøe et al. 1989)
USA (4)	*C. punctulata* (Læssøe et al. 1989), C. *signata* (Læssøe et al. 1989), C. *texensis* (Rogers et al. 2008), C. *tinctor* (Læssøe et al. 1989)
Brazil (3)	*C. bilabiata* (Spegazzini 1889), *C. flosculosa* (Læssøe et al. 1989), C. *sulcata* (Lloyd 1918)
Malaysia (2)	*C. malaysianensis* (Whalley et al. 1999), *C. selangorensis* (Whalley et al. 1996)
Venezuela (2)	*C. punctidisca* (Læssøe et al. 1989), C. *venezuelensis* (Dennis 1970)
Colombia (1)	*C. hainesii* (Læssøe et al. 1989)
Martinique (FWI)(1)	*C. lechatii* (Fournier 2022)
Thailand (1)	*C. malaysianensis* var. *macrospora* (Vasilyeva et al. 2012)
Trinidad (1)	*C. macrospora* (Hastrup and Læssøe 2009)

During 2007 to 2021, two of us (JF and CL) had been collecting in French Guiana for 1–2 weeks every year, accumulating material of predominantly Hypocreales and Xylariales. *Camillea* was often well represented and, aside from the more commonly encountered species with upright stromata, those with applanate stromata and apparently absent in the monograph by Læssøe et al. (1989) required more work to be identified, most often tentatively. Five recently collected new species described in this study are surprisingly documented from French Guiana for the first time since the Leprieur and Montagne time, highlighting the importance of field work and sampling efforts before assessing the richness and distribution of fungal species. This is especially true for environments such as the Guianese forests, in which ascomycetes are both highly species-rich and still little investigated.

Professor Rogers published his first paper on *Camillea* in 1968 and since then has continued to take a keen interest in this intriguing genus, describing many new species and adding substantially to the knowledge of *Camillea* (Læssøe et al. 1989; Rogers 1968, 1975a, b, 1977, 1980; Rogers and Dumont 1979; Rogers et al. 1991, 2000, 2002, 2008; San Martín and Rogers 1993). In recognition of his work and profoundly inspiring influence, we are pleased to help honor his memory by dedicating this work and a new species bearing his name.

## Methods

### Fungal materials and morphological observations by light microscopy and scanning electron microscopy

Stromata of *Camillea* species were collected in French Guiana by JF and CL during 2007 to 2021. Specimens were deposited at the herbarium of Biodiversity Research Museum, Academia Sinica, Taiwan (HAST). Cultures obtained from single spore isolation on PDA were incubated at 25 °C, and descriptions were made at 3 wks incubation.

Measurements of asci and ascospores were made in water and ascospores measurements processed with the free software Piximetre 5.2 (http://ach.log.free.fr/Piximetre/). In the formula given by this software the values in brackets represent the extreme values (20%) that are not taken into account for the calculation, N represents the number of ascospores measured, Q the quotient length/width, Me the mean values of length × width and Qe the mean value of quotient length/width. Measurements of stromata account for all material available, the values in brackets accounting for unusually small or large stromata. At least 10 asci were measured for each specimen.

See Læssøe et al. (1989) for terminology of ostiolar areas. Specific notes on ascal apical structures, content of paraphyses, and sectioning of carbonaceous stromata can be found in Fournier (2022). They introduce the important notion of a bipartite apical apparatus made up of an upper inamyloid structure, lenticular to more massive, stained blue with ink or grey with chlorazol black, which we call pulvillus; the lower part, which stains deep blue with Melzer’s reagent, is called the subapical apparatus.

Abbreviations e.g. and SDS refer respectively to “for example” and to Sodium dodecyl sulfate.

Observation by scanning electron microscopy (SEM) was performed as follows. Ascospores were released from asci and subjected to the following procedures prior to critical point drying. The fungal materials were loaded on a 0.45-µm membrane, and fixed in 2.5% glutaraldehyde and 4% paraformaldehyde in 0.1 M sodium phosphate buffer of pH 7.0 for 2 min with 250 W microwave (microwave biological sample preparation system, Pelco BioWave Pro+). The fixed samples were rinsed with a buffer for 1 min three times with 250 W microwave, subsequently dehydrated in an ethanol series with 100 W microwave, and, as a final step, subjected to critical point drying with Leica EM CPD300 critical point dryer. The dehydrated samples were coated in a Hitachi E-1010 ion sputter and observed with a FEI Quanta 200 scanning electron microscope at 20 KV.

### Molecular methods

Sequences of ITS and LSU were mainly obtained from specimens. Total DNA was extracted from perithecial tissue and their surrounding stromatal tissue. The tissues were ground using stainless steel beads of 1, 2, and/or 5 mm within a polypropylene microvial in a Mini-Beadbeater-16 (BioSpec Products, Bartlesville, OK) for various durations ranging from 30 s to 2 min. The DNA extraction was then performed using the TANBead Fungal Nucleic Acid Extraction Kit and TANBead Nucleic Acid Extractor (Taiwan Advanced Nanotech, Taipei, Taiwan), following the manufacturer’s protocol. Polymerase chain reaction (PCR) amplifications and sequencing of nuc rDNA internal transcribed spacers (ITS1-5.8 S-ITS2 = ITS) followed Hsieh et al. (2009), whereas those of nuc rDNA large subunit (LSU) followed Vilgalys and Hester (1990) with primer sets of LR0R/LR5, LR0R/LR6, or LR0R/LR7 and a program of 94 °C for 5 min, 94 °C for 1.3 min, 48 °C for 1.5 min, 72 °C for 2 min, 72 °C for 10 min and 40 repeated cycles.

### Phylogenetic analyses

*Camillea* species included in the phylogenetic analyses are listed in Table 2. Sequences of ITS and LSU were separately aligned using the program Clustal X 1.81 (Thompson et al. 1997) with the “gap penalty” set to 10 and “gap extension penalty” set to 0.2, and were improved manually. The concatenated dataset of ITS and LSU (ITS-LSU dataset) of studied species was used to generate the Maximum-Likelihood (ML) trees using RAxML analysis ver. 8.2.10 (Stamatakis 2014) with rapid bootstrap support and 1000 replicates of bootstrap test. Bayesian Inference (BI) analyses were performed with MrBayes ver. 3.2.6 (Ronquist et al. 2012) using a Markov Chain Monte Carlo (MCMC) algorithm. Four MCMC chains (one cold and three heated) were run for one million generations with the trees sampled every 100 generations. The first 25% trees were excluded as the burn-in phases of the analysis, and the posterior probability values were estimated with the 75% remaining trees. Models of evolution for ML or BI trees were defined by MrModeltest 2.4 (Nylander 2004). The consensus trees were viewed in FigTree ver. 1.4.4 (http://tree.bio.ed.ac.uk/software/figtree/). *Xylaria fimbriata* C. G. Lloyd was used as the outgroup.

**Table 2:** Taxa included in the phylogenetic analyses. Note that sequences of those taxa in boldface were generated in this study

Taxon	Origin	Collecting data	GenBank accession number	
			ITS	LSU
*C. broomeana*	China	*Li, Q.-R. 2020FCGY11* (Li et al. 2021)	MW854657	MW854663
*C. cribellum*	French Guiana	Holotype (the present study)	**OQ871491**	**OQ871465**
*C. cyclisca*	French Guiana	Maripasoula, Saül, trail head toward Mont La Fumée, 3.628084 N, 53.207105 W, disturbed rainforest, 230–250 m, on dead corticated branchlet, 21 Aug 2018, *Fournier, J. GYJF18051* (HAST 145987)	**OQ871482**	**OQ871470**
*C. fossulata*	French Guiana	Maripasoula, Saül, trail head to Roche Bateau, 3.620498 N, 53.199309 W, disturbed mesophilic rainforest, ca. 240 m, on dead corticated branchlet, 22 Jun 2019, *Fournier, J. GYJF19180* (HAST 145988)	**OQ871483**	**OQ871471**
*C. fusiformis*	French Guiana	Maripasoula, Saül, trail to Sentier des Gros Arbres, Crique Grand-Fossé, 3.617198 N, 53.209029 W, disturbed mesophilic rainforest, ca. 210 m, on dead corticated branch ca. 20 mm diam, 1 Apr 2021, *Fournier, J. GYJF21346* (HAST 145989)	**OQ871484**	**OQ871472**
*C. heterostoma* var. *heterostoma*	French Guiana	Maripasoula, Saül, trail head toward Sentier des Gros Arbres, 3.620589 N, 53.208547 W, disturbed rainforest, ca. 210 m, on dead corticated branch, 22 Aug 2018, *Fournier, J. GYJF18086* (HAST 145990)	**OQ871485**	**OP919599**
*C. heterostoma* var. *microspora*	French Guiana	Maripasoula, Saül, trail head to Sentier des Gros Arbres, 3.6201 N, 53.207989 W, ca. 210 m, on dead corticated branch, 28 Mar 2021, *Fournier, J. GYJF21198* (HAST 145991)	**OP595819**	**OQ871473**
*C. heterostomoides*	French Guiana	Holotype (the present study)	**OP595160**	**OQ871466**
*C. labellum*	French Guiana	Maripasoula, Saül, trail head to Sentier des Gros Arbres, 3.6201 N, 53.207989 W, ca. 210 m, on dead corticated branch, 28 Mar 2021, *Fournier, J. GYJF21200* (HAST 145992)	**OQ871486**	**OQ871474**
*C. leprieurii* (applanate form)	French Guiana	Maripasoula, Saül, Sentier des Gros Arbres, crique Grand Fossé, 3.617198 N, 53.209029 W, disturbed mesophilic rainforest, ca. 210 m, on dead corticated branch, 1 Apr 2021, *Fournier, J. GYJF21338-1* (HAST 145993)	**OQ871487**	**OQ871475**
*C. leprieurii* (applanate form)	(to be added)	Régina, banks of Approuague in a *Theobroma* plantation next to the village, on dead corticated branch, 28 Apr. 2010, Lechat C., *CLLG10045* (LIP)	**OP595154**	**OP919569**
*C. leprieurii* (upright form)	French Guiana	Maripasoula, Saül, Sentier des Gros Arbres, crique Grand Fossé, 3.617198 N, 53.209029 W, disturbed mesophilic rainforest, ca. 210 m, on dead corticated branch, 1 Apr 2021, *Fournier, J. GYJF21338-2* (HAST 145994)	**OQ871488**	**OQ871476**
*C. macrospora*	French Guiana	Maripasoula, Saül, trail head to Roche Bateau, airfield edge, 3.620754 N, 53.200605 W, disturbed mesophilic rainforest, ca. 240 m, on dead corticated branch, 23 Jun 2019, *Fournier, J. GYJF19228* (HAST 145995)	**OQ871489**	**OQ871477**
*C. nitida*	French Guiana	From holotype (the present study)	**OQ871492**	**OQ871467**
*C. obularia*	Puerto Rico	*ATCC093* (Wendt et al. 2018)	KY610384	KY610429
*C. obularia*	French West Indies	Martinique: Sainte-Marie: La Philippe, Trou-Mulet, 14.803724 N, 61.00407 W, ca. 30 m, coastal mesophilic rainforest, on dead corticated trunk, 2 Aug 2016, *Fournier, J. MJF16091* (LIP)	**OP587265**	**OP919567**
*C. rogersii*	French Guiana	From holotype (the present study)	**OP595158**	**OP729385**
*C. saulensis*	French Guiana	From holotype (the present study)	**OQ871493**	**OQ871468**
*C. hyalospora*	French West Indies	Martinique: Le Prêcheur, Anse Couleuvre, 14.840197 N, 61.216688 W, ca. 20 m, coastal mesophilic forest, on dead corticated branch, 4 Aug. 2016, *Fournier, J. MJF16119* (LIP)	**OP595153**	**OP919602**
*C. stellata*	French Guiana	Maripasoula, Saül, village, next to Maison du Parc, 3.622504 N, 53.208516 W, ca. 210 m, on dead corticated branch in a pile of dead branches of *Mangifera indica*, 24 Aug 2018, *Fournier, J. GYJF18180* (HAST 145996)	**OQ871490**	**OQ871478**
*C. tinctor*	French West Indies	Martinique, Chemin du Saut des Trois Cornes, on dead wood, 22 Aug 2005, *Lechat, C. CLL1034* (HAST 145997)	JX507806	**OQ871479**
*Xylaria fimbriata*	French West Indies	*Lechat, C. CLL5010* (Hsieh et al. 2010)	GU324753	**OQ871480**

## Results and discussion

### Phylogenetic analyses

**Fig. 1:**
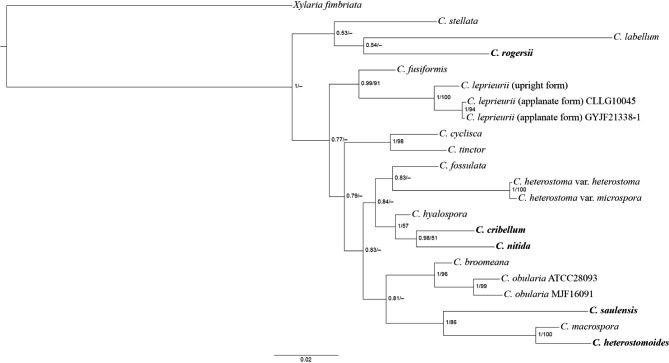
Phylogenetic tree generated by BI analysis from the ITS-LSU dataset. The newly described species are in boldface. Numbers at internodes represent posterior probability values and are immediately followed by bootstrap values greater than 50 generated by ML analysis

Sequences available for inferring phylogenetic relationships between *Camillea* species are scarce, mainly from ITS and LSU regions (Table 2). BI and ML trees based on the ITS-LSU dataset displayed similar topologies, showing that the five *Camillea* species described as new herein from French Guiana were distinct from those known species that have available sequences for comparison (Fig. 1). In general, ITS sequences were more variable than LSU sequences among analyzed taxa. When taxa shared highly similar ITS sequences, we are inclined to consider them in synonymy. *Camillea heterostoma* (Mont.) Læssøe, J. D. Rogers & Whalley var. *heterostoma* and *C*. *heterostoma* var. *microspora* J. D. Rogers, F. San Martín & Y.-M. Ju happen to be such a case, where their ITS sequences shared a 99.82% similarity; we thus consider them in synonymy. *Camillea broomeana* (Berk. & M. A. Curtis) Læssøe, J. D. Rogers & Whalley was treated as a synonym of *C*. *obularia* by Rogers et al. (1991), but the species identified as *C*. *broomeana* by Li et al. (2021) from China shared only a 94.35% similarity at ITS with *C*. *obularia* from the Caribbean but a 99.87% similarity at LSU, suggesting that there may be a taxon similar to *C*. *obularia* existing in Asia. *Camillea leprieurii* and *Hypoxylon melanaspis* (Mont.) Mont. have upright and applanate stromata, respectively, but have long been treated as two forms of a species (Læssøe et al. 1989). Our SEM study also shows different ascospore ornamentation patterns (unpublished data). The two forms shared a 97.83% similarity at ITS but a 99.32% similarity at LSU. More collections should be studied before a final conclusion can be drawn.

### Taxonomy

**Fig. 2:**
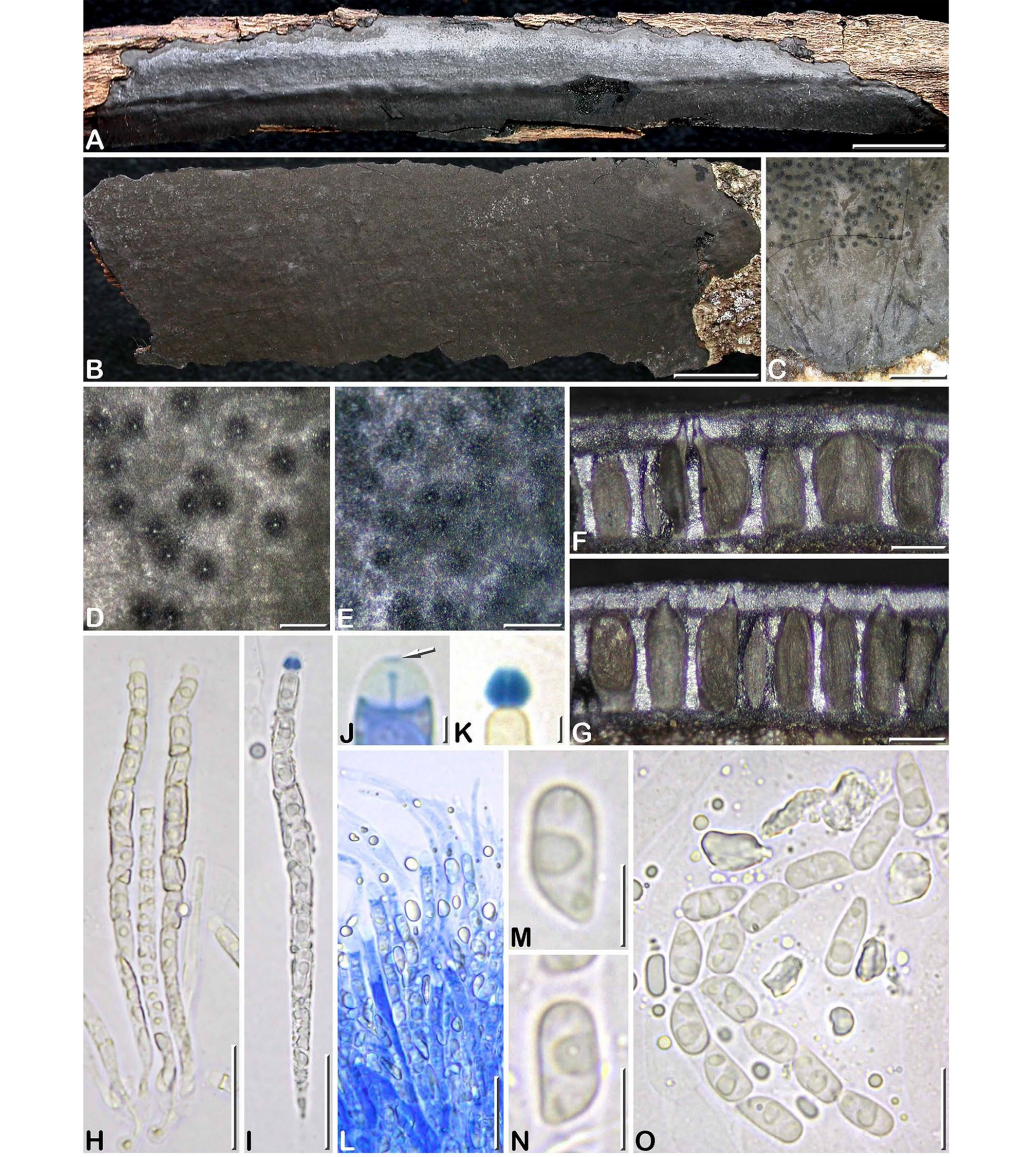
*Camillea cribellum*. (A, E, F, J–O. GYJF 18137 from holotype; B–D, G–I from paratype CLL 0820). **A** Habit of a narrowly elongate stroma showing a slightly shiny surface; **B** Fragmentary widely spread stroma with dull black surface; **C** Grey margin of a stroma and fertile part finely dotted with black; **D, E** Surface of two different stromata in close-up, showing blackish halos around ostiolar openings; **F, G** Vertical sections of two different stromata; **H, I** Asci showing oily content around ascospores, in 1% SDS and Melzer’s reagent respectively; **J** Ascus apical apparatus showing a minute apical pulvillus slightly stained by blue Pelikan ink (arrow); **K** Rhomboid subapical apparatus stained by Melzer’s reagent; **L** Hymenium stained by blue Pelikan ink showing paraphyses tapering above asci and containing large refractive guttules; **M, N** Ascospores in side view; **O** Variously shaped ascospores interspersed with oily guttules and amorphous refractive bodies (M-O in 1% SDS). Scale bars: A, B = 10 mm; C = 1 mm; D-G = 0.2 mm; H, I, L = 20 μm; J, K = 2 μm; M, N = 5 μm; O = 10 μm

**Fig. 3:**
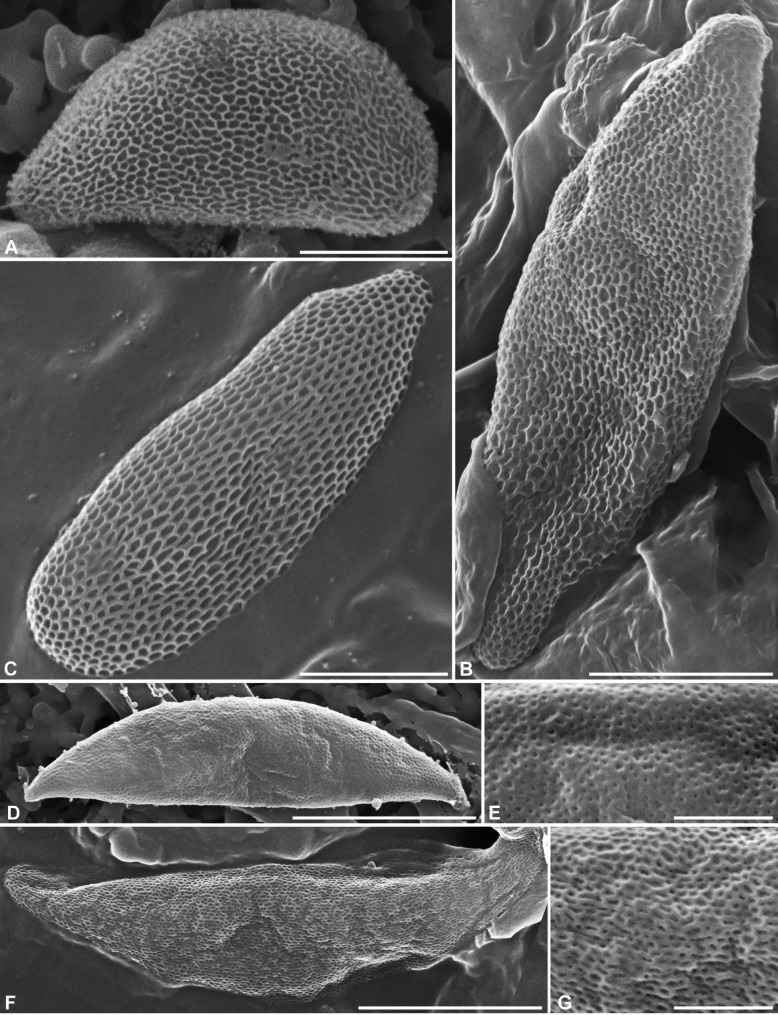
Ascospores of *Camillea* species by SEM. **A ***C*. *cribellum* (from holotype). **B ***C*. *heterostomoides* (from holotype). **C ***C*. *nitida* (from holotype). **D, E ***C*. *rogersii* (from holotype). **F, G ***C*. *saulensis* (from holotype). Scale bars: B, D, F = 5 μm; A, C = 2.5 μm; E, G = 1 μm

***Camillea cribellum*** J. Fourn. & Y.-M. Ju sp. nov. Figs. 2, 3A, Table 3.

**Table 3:** Ascospore and subapical apparatus dimensions in four collections of *C. cribellum* from various neotropical origins

Collections numbers	Ascospore measurements with extreme values in parentheses	Q = quotient l/w, N = number of measurements	Mean values	Subapical apparatus h × w µm, N = 25
*GYJF 18137* holotype FG	(6.8–)7.3–9.0(–9.3) × (3.4–)3.5–4.3(–4.5) µm	Q = (1.7–)1.9–2.3(–2.6) N = 60	Me = 8.1 × 3.9 µm Qe = 2.1	Me = 2.8 × 3.4
*CLL 8020* FG	(7.2–)7.4–8.6(–9.6) × (3.3–)3.6–4.4(–4.6) µm	Q = (1.6–)1.8–2.2(–2.5) N = 60	Me = 8 × 4 µm Qe = 2	Me = 2.2 × 3.2
*CLL 2310* Guadeloupe	(7.2–)7.8–8.9(–9.8) × (3.5–)3.8–4.4(–4.6) µm	Q = (1.7–)1.8–2.2(–2.5) N = 60	Me = 8.3 × 4.1 µm Qe = 2	
*MJF07271-2* Martinique	(6–)6.8–8(–8.8) × (3–)3.3–4.1(–4.5) µm	Q = (1.7–)1.8–2.3(–2.9) N = 60	Me = 7.4 × 3.7 µm Qe = 2	
*MJF 14055* Martinique	(7.2–)7.6–8.8(–10.1) × (3.4–)3.6–4.3(–4.8) µm	Q = (1.7–)1.8–2.3(–2.6) N = 60	Me = 8.2 × 4 µm Qe = 2.1	
*MJF 14118* Martinique	(6.5–)7.4–8.9(–9.3) × (3–)3.5–4.2(–4.5) µm	Q = (1.7–)1.9–2.3 (–3) N = 60	Me = 8 × 3.8 µm Qe = 2.1	Me = 2.3 × 3
*MJF 16196* Martinique	(6.5–)7–8.4(–9.2) × (3–)3.5–4(–4.2) µm	Q = (1.6–)1.8–2.3(–3) N = 60	Me = 7.6 × 3.8 µm Qe = 2	
cumulated values	(6.5–)7–9(–10.1) × (3–)3.5–4.4(–4.8) µm	Q = (1.6–)1.8–2.3(–3) N = 420	Me = 7.9 × 3.9 µm Qe = 2	Me = 2.4 × 3.2

#### MycoBank MB 848864

##### Typification

FRENCH GUIANA: Maripasoula, Saül, village, next to Maison du Parc, 3.622504 N, 53.208516 W, ca. 210 m, on a dead corticated branch in a pile of dead branches of *Mangifera indica*, 24 Aug. 2018, *Fournier, J.* GYJF 18137 (HAST 145953 Holotype), GenBank: ITS = OQ871491, LSU = OQ871465.

##### Etymology

From Latin *cribellum* = sieve, for the stromatal surface evenly dotted with minute umbilicate ostioles.

##### Diagnosis

Differs from all known species of *Camillea* by the combination of thinly applanate stromata up to 0.5 mm thick with plane surface evenly dotted with minute umbilicate ostioles and rectangular ascospores attenuated at one end, 7.9 × 3.9 μm on average.

Stromata erumpent through bark, (5–)20–90 mm long × (3–)5–30 mm wide, occasionally more widely spread on larger branches and fragmentary, 0.45–0.5 mm thick, applanate, narrowly elongate to irregularly ellipsoid, with a narrow, dark grey to dull black sterile margin; surface dark grey to most often dull black, occasionally slightly shiny black, plane to slightly undulate according to the irregularities of the underlying bark; subsurface crust 100–120 μm thick, carbonaceous; interperithecial tissue entirely carbonaceous, black, and subperithecial tissue reduced to a thin black carbonaceous layer. Perithecia flask-shaped, laterally and basally flattened, 0.29–0.34 mm high × 0.13–0.20 mm diam, opening individually through central to most often eccentric ostiolar necks. Ostioles uniformly distributed, inconspicuous, appearing as minute grey to black dots ca. 15–25 μm diam, at surface level or faintly umbilicate, frequently surrounded by a blackish halo 80–100 μm diam contrasting with the paler background, occasionally located in faint, shallow, ill-defined depressions.

Paraphyses hyphal, thin-walled, remotely septate, 4–6 μm wide at base, tapering to 1.5–2 μm wide with conspicuous refractive guttules; perithecial content colorless, composed of amorphous refractive bodies before maturation of asci, which gradually vanish. Asci narrowly cylindrical, with eight uniseriately arranged, slightly overlapping ascospores, 76–88 μm in total length × 5–6 μm, including an attenuated lower end up to 20 μm long, with conspicuous oily content filling the ascus between ascospores and fragmented into small oily guttules when ascospores are released; apical apparatus 3.6–4.2 μm high, bipartite, comprised of a rhomboid subapical apparatus 2.1–3.1 × 3–3.6 μm (Me = 2.4 × 3.2 μm, N = 75), bluing in Melzer’s reagent and a reduced inamyloid upper part with a minute, discoid apical pulvillus faintly stained blue by blue Pelikan ink.

Ascospores (6.5–)7–9(–10.1) × (3–)3.5–4.4(–4.8) µm, Q = (1.6–)1.8–2.3(–3), N = 420 (Me = 7.9 × 3.9 μm, Qe = 2), in side view rectangular with a beveled, narrowly rounded end and the other end broadly rounded, bullet-shaped in dorsal or ventral view, light yellowish, consistently oriented with the beveled end toward the base of the ascus, with 1–2 oily droplets; no germ slit visible; epispore smooth by LM, angular reticulate-poroid with relatively thin septa by SEM (Fig. 3A).

##### Cultures and anamorph

Unknown.

##### Additional specimens examined (paratypes)

FRENCH GUIANA: Sinnamary, Saint-Elie botanical trail, 5.292020 N, 53.05262 W, mesophilic rainforest, on a dead corticated branchlet, 28 Apr. 2008, *Lechat, C.* CLL8020 (HAST 145954). FRENCH WEST INDIES: GUADELOUPE: Sainte-Rose, Sofaïa, path to Saut des Trois Cornes, 16.290489 N, 61.727007 W, mesophilic rainforest, 3 Sept. 2004, *Lechat, C.* CLL 2310 (HAST 145955). MARTINIQUE: Fort-de-France, Absalon, track to Plateau Michel, hygrophilic rainforest, 14.676801 N, 61.096398 W, dead corticated branch, 5 Jun. 2014, *Fournier, J*. MJF 14055 (HAST 145956); Le Marigot, Habitation Denel, Pérou forest road, track to Morne Bellevue, hygrophilic rainforest, 14.741993 N, 61.053561 W, ca. 530 m, dead corticated branch, 10 Jun. 2014, *Fournier, J*. MJF14118 (HAST 145958); Fort-de-France, forest track of Fond-Baron, hygrophilic rainforest, 14.678116 N, 61.091136 W, ca. 400 m, dead corticated branch, 10 Aug. 2016, *Fournier, J*. MJF 16196 (HAST 145957); Le Prêcheur, Anse Couleuvre, mesophilic coastal rainforest, 14.840537 N, 61.217306 W, on a dead corticated branchlet, 2 Sep 2007, *Fournier, J*. MJF 07271-2 (largely immature) (HAST 145959).

##### Known distribution

French Guiana; French West Indies (Guadeloupe, Martinique).

**Notes.** The stromata of most of applanate species of *Camillea* exhibit distinctive morphological variations regarding the distribution and differentiation of ostiolar structures which vary from pits to more or less prominent papillae or are associated with annulate rims or furrows, depressions or bumps on stromatal surface. *Camillea cribellum* is set apart by its inconspicuous, uniformly distributed ostioles, in relation with perithecia opening individually, and so inconspicuous that they need to be observed with a stereomicroscope at high magnification. Their presence is underlined by diffuse black spots surrounding them on stromatal surface but they definitely lack associated rims or furrows.

In some of the collections examined, the surface may appear in places uneven due to shallow ill-defined depressions surrounding the ostiolar openings. This recalls *C. fossulata* (Mont.) Læssøe, Rogers & Whalley and *C. mexicana* F. San Martin & J. D. Rogers, two species that also feature similar ascospores and to which *C. cribellum* is likely closely related. Both primarily differ from *C. cribellum* by finely papillate ostioles, located individually in small rounded depressions ca. 200 μm diam for *H. fossulata* or clustered by 1–4 in larger, more polygonal, coalescent depressions for *C. mexicana*.

The ascospore ornamentation revealed by SEM sets *C. cribellum* apart from other known species with rectangular ascospores attenuated at one end, i.e., *C. fossulata* (San Martín and Rogers 1993), *C. hainesii* (J. D. Rogers & Dumont) Læssøe, J. D. Rogers & Whalley (Rogers and Dumont 1979), *C. nitida* (this paper), *C. mexicana* (San Martín and Rogers 1993), *C. punctidisca* (J. D. Rogers) Læssøe, J. D. Rogers & Whalley (Læssøe et al. 1989) and *C. punctulata* (Berk. & Ravenel) Læssøe, J. D. Rogers & Whalley (Læssøe et al. 1989) by a more angular and more loosely reticulate pattern with thinner septa.

Other known species with such small rectangular ascospores are difficult to distinguish by their ascospore dimensions that are often overlapping, thus ostiolar morphology remains the more discriminating character when SEM data on ascospore ornamentation are not available.

See comments on *C. nitida* in this paper.

**Fig. 4:**
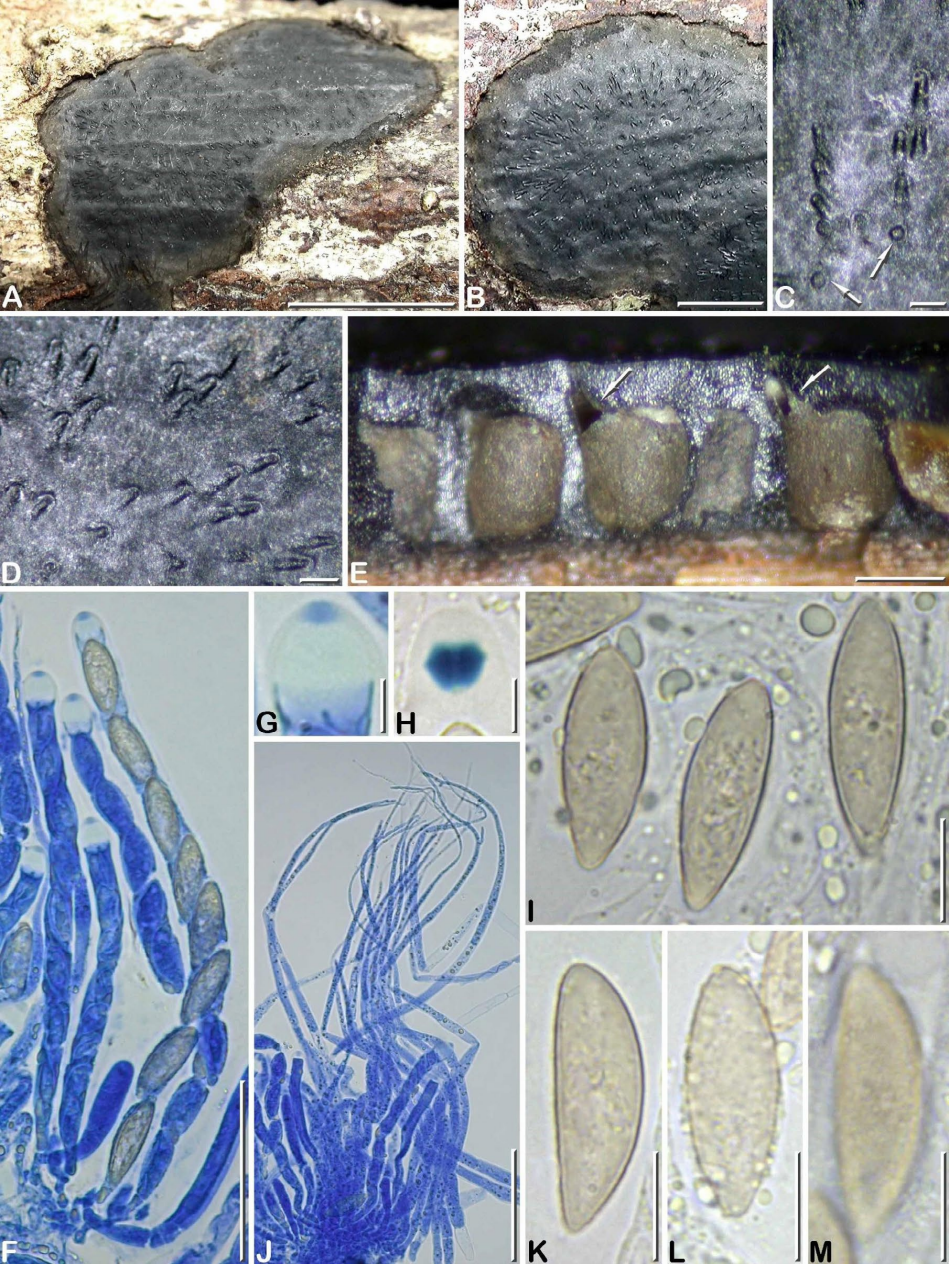
*Camillea heterostomoides* (from holotype). **A, B** Habit of stromata on host surface; **C, D** Stromatal surface in close-up showing rounded (arrows) to elongate ostiolar depressions; **E** Stroma in vertical section showing carbonaceous tissue surrounding the perithecia opening through eccentric ostiolar canals (arrows); **F** Immature and mature asci, in blue Pelikan ink; **G** Ascus apex in blue Pelikan ink, showing an apical pulvillus stained blue; **H** Ascus apex showing the rhomboid subapical apparatus bluing in Melzer’ reagent; **I** Variously shaped ascospores; **J** Bundle of paraphyses, in blue Pelikan ink; **K** Ascospore in side view; **L** Freshly released ascospores coated with oily guttules; **M** Ascospore in side view with focus on epispore showing an obscure reticulate ornamentation (I, K-M in 1% SDS). Scale bars: A = 5 mm; B = 2 mm; C, D, E = 0.2 mm; F, J = 50 μm; G, H = 5 μm; I, K–M = 10 μm.

**Fig. 5:**
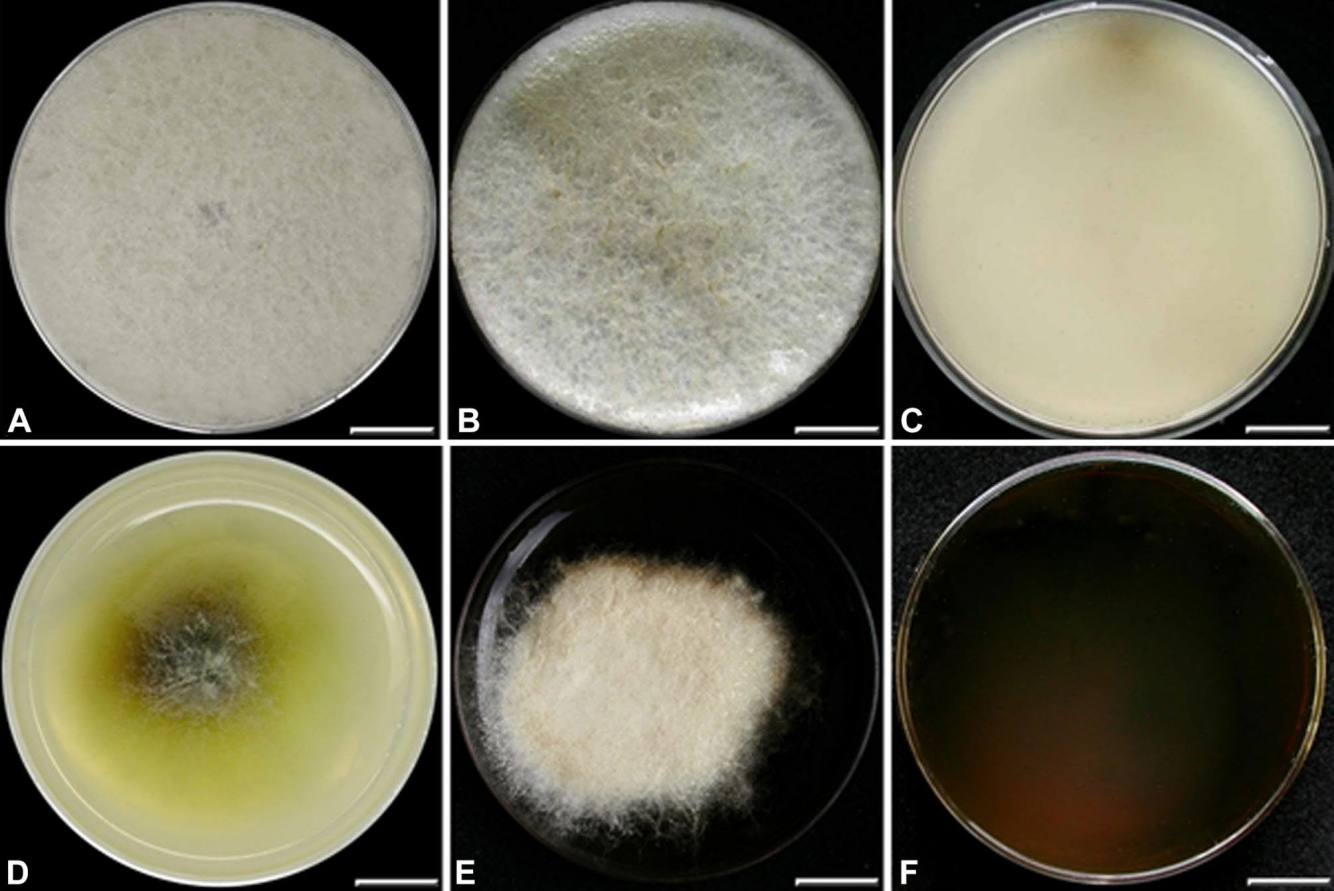
Cultures of *Camillea* species. **A–C ***C. heterostomoides* (from holotype). **A** Colony at 1 wk; **B** Colony at 3 wk; **C** Reverse at 3 wk; **D–F ***C. rogersii* (from holotype). **D** Colony at 1 wk; **E** Colony at 3 wk; **F** Reverse at 3 wk. Scale bars: A–F = 1 cm.

***Camillea heterostomoides*** J. Fourn. & Y.-M. Ju sp. nov. Figs. 3B, 4,  5A–C.

#### MycoBank MB 848865

##### Typification

FRENCH GUIANA: Maripasoula, Saül, trail head to Sentier des Gros Arbres, 3.6201 N, 53.207989 W, disturbed mesophilic rainforest, ca. 210 m, on a dead corticated branchlet, 27 Mar. 2021, *Fournier, J.* GYJF 21170-2 (HAST 145960 holotype), ex-type culture BRFM 3480, GenBank: ITS = OP595160, LSU = OQ871466.

##### Etymology

From *heterostoma* combined with the Greek suffix –oïdes = -like, for its resemblance with *C. heterostoma*.

##### Diagnosis

Differs from *C. heterostoma* by perithecia opening individually and significantly larger ascospores 21.2–26.3 × 6.5–7.4 μm vs. 10.1–14.5 × 4.7–6.6 μm.

Stromata erumpent through bark, applanate, orbicular 5–6 mm diam to irregularly ellipsoid 11–14 mm × 6–11 mm diam by coalescence of two stromata, 0.4–0.5 mm thick; surface dull black with olivaceous grey sterile margins 0.5–1 mm wide, smooth to faintly wrinkled, finely roughened by minute, widely scattered narrowly wedge-shaped ostiolar depressions 170–210 μm long × 70–85 μm wide, radiating outward often in short linear rows, with outward end curving upward and slightly raising stromatal surface to form a low rim, at center of the fertile part of the stroma occasionally rounded and 50–80 μm diam; subsurface crust 85–130 μm thick, black, carbonaceous; interperithecial tissue black, carbonaceous, brittle. Perithecia subglobose to cuboid, laterally and basally flattened, 0.30–0.35 mm high × 0.20–0.25 mm diam, opening individually through central to most often eccentric ostiolar necks, seated on a thin, black, carbonaceous basal layer; underlying bark tissue stained vivid orange. Ostioles inconspicuous, opening at the outermost part of the rim through a grey disc 30–40 μm diam.

Paraphyses copious, hyphal, thin-walled, remotely septate, sparsely and minutely guttulate, 4.0–6.0 μm wide at base, tapering to 1.0–1.5 μm wide above asci; perithecial content colorless. Asci cylindrical, subsessile, with (6–)8 uniseriately arranged, slightly overlapping ascospores, 160–190 μm long × 7.0–10 μm wide, with sparse oily content around maturing ascospores, with a bipartite apical apparatus 7.0–10 μm high, comprised of a rhomboid subapical apparatus 4.1–4.6 × 4.5–5.2 μm (Me = 4.3 × 4.8 μm, N = 25) with sharp lateral rims, bluing in Melzer’s reagent and an inamyloid upper part readily swelling in water, with an apical pulvillus 1.7 × 3.3 μm on average, stained blue by blue Pelikan ink.

Ascospores (19.7–)21.2–26.3(–30.3) × (6.0–)6.5–7.4(–8.9) µm, Q = (2.7–)3.0–3.8(–4.2), N = 60 (Me = 23.7 × 7.0 μm, Qe = 3.4), fusiform slightly inequilateral, heteropolar with obtuse upper end and narrowly rounded to subacute lower end, yellowish to light yellow-brown; epispore smooth to obscurely reticulate by LM, reticulate poroid by SEM (Fig. 3B).

##### Cultures and anamorph

Colonies reaching the edge of Petri dish within 10 days; mycelium white, appressed, cottony, superficially fluffy, becoming light orange brown in places with age; reverse colorless; medium unstained; odor faint, sweetish. No conidiogenesis observed after 6 weeks.

##### Known distribution

French Guiana, known only from the type collection.

**Notes.** This collection is sparse and unfortunately largely depauperate. However, the presence of one fertile stroma allowed for a successful culture yielding DNA sequences, and provided sufficient microscopic evidence to introduce this new *Camillea* species. The most closely resembling taxa to be compared with *C*. *heterostomoides* were *C. heterostoma* and its variety *microspora*. Ascospore measurements carried out on two specimens referable to the variety *microspora* because of slightly smaller ascospores showed intermediate values between those of the typical variety and those from the protologue of the variety *microspora* (Table 4). In the absence of further differential morphological characters and based on the high similarity of their ITS sequences, both taxa are regarded as synonyms.

**Table 4:** Ascospore and subapical apparatus dimensions of *C. heterostomoides* compared with those of *C. heterostoma* and relatives sorted in descending order of ascospore dimensions

collections numbers	Ascospore measurements with extreme values in parentheses	Q = quotient l/w, N = number of measurements	Mean values	Subapical apparatus h × w µm, N = 25
* C. heterostomoides* holotype	(19.7–)21.2–26.3(–30.3) × (6.0–)6.5–7.4(–8.9) µm	Q = (2.7–)3.0–3.8(–4.2), N = 60	Me = 23.7 × 7.0 μm Qe = 3.4	Me = 4.3 × 4.8
*Hypoxylon heterostomum* var. *macrosporum* (Miller 1961)	25–30 × 8–10 μm	–	Me = 27.5 × 8.0 μm Qe = 3.4	–
*C. macrospora GYJF 19228*	(16.4–)18.1–21.5(–23.0) × (6.1–)6.9–7.8(–8.2) µm	Q = (2.2–)2.4–3.0(–3.4), N = 60	Me = 19.6 × 7.3 μm Qe = 2.7	Me = 4.1 × 6.0
* C. heterostoma* (Læssøe et al. 1989)	(10.8–)11.3 × 15.5(–16.5) µm	N = 50	Me = 13.8 × 6.0 μm Qe = 2.3	Me = 3.3 × 5.5
* C. heterostoma GYJF 18086*	(10.8–)12.0–14.5 (–16.0) × (5.0–)5.4–6.1(–6.4) µm	Q = (2.0–)2.1–2.6(–2.9), N = 60	Me = 13.0 × 5.7 μm Qe = 2.3	Me = 2.9 × 4.2
* C. heterostoma* var. *microspora GYJF 21198*	(10.1–)10.8–12.8(–14.1) × (4.5–)4.9–5.4(–5.9) µm	Q = (1.9–)2.1–2.5(–2.7), N = 60	Me = 11.8 × 4.8 μm Qe = 2.3	–
*C. heterostoma* var. *microspora GYJF 21300*	(7.6–)10.1–12.3(–14.2) × (4.5–)4.7–6.2(–6.5) µm	Q = (1.3–)1.7–2.4(–2.7), N = 60	Me = 11.3 × 5.5 μm Qe = 2.0	Me = 2.8 × 4.3
* C. heterostoma* var. *microspora* (Rogers et al. 2002)	9–12 × 4.5–5 μm	–	Me = 10.5 × 5.2 μm Qe = 2.5	–

*Camillea heterostomoides* is primarily distinguished from *C. heterostoma* by thinner stromata less than 0.5 mm thick, smaller ostiolar rims and significantly larger ascospores (Table 4) (Læssøe et al. 1989; Miller 1961; Rogers et al. 2002; Hastrup and Læssøe 2009; JF unpublished observations). *Camillea macrospora* (J.H. Miller) Hastrup & Læssøe (formerly *H. heterostomum* var. *macrosporum* J. H. Miller) has similar but larger ascospores than *C. heterostomoides* and *C. heterostoma*. Unlike these two species, *C. macrospora* features ostioles that are located in deep oval depressions (Hastrup and Læssøe 2009). It is noteworthy that the bark tissue underlying the stromata of both *C. heterostomoides* and *C. macrospora* is strikingly stained orange, recalling a feature often but not consistently present in collections assigned to *C. tinctor* (Berk.) Læssøe, J. D. Rogers & Whalley.

**Fig. 6:**
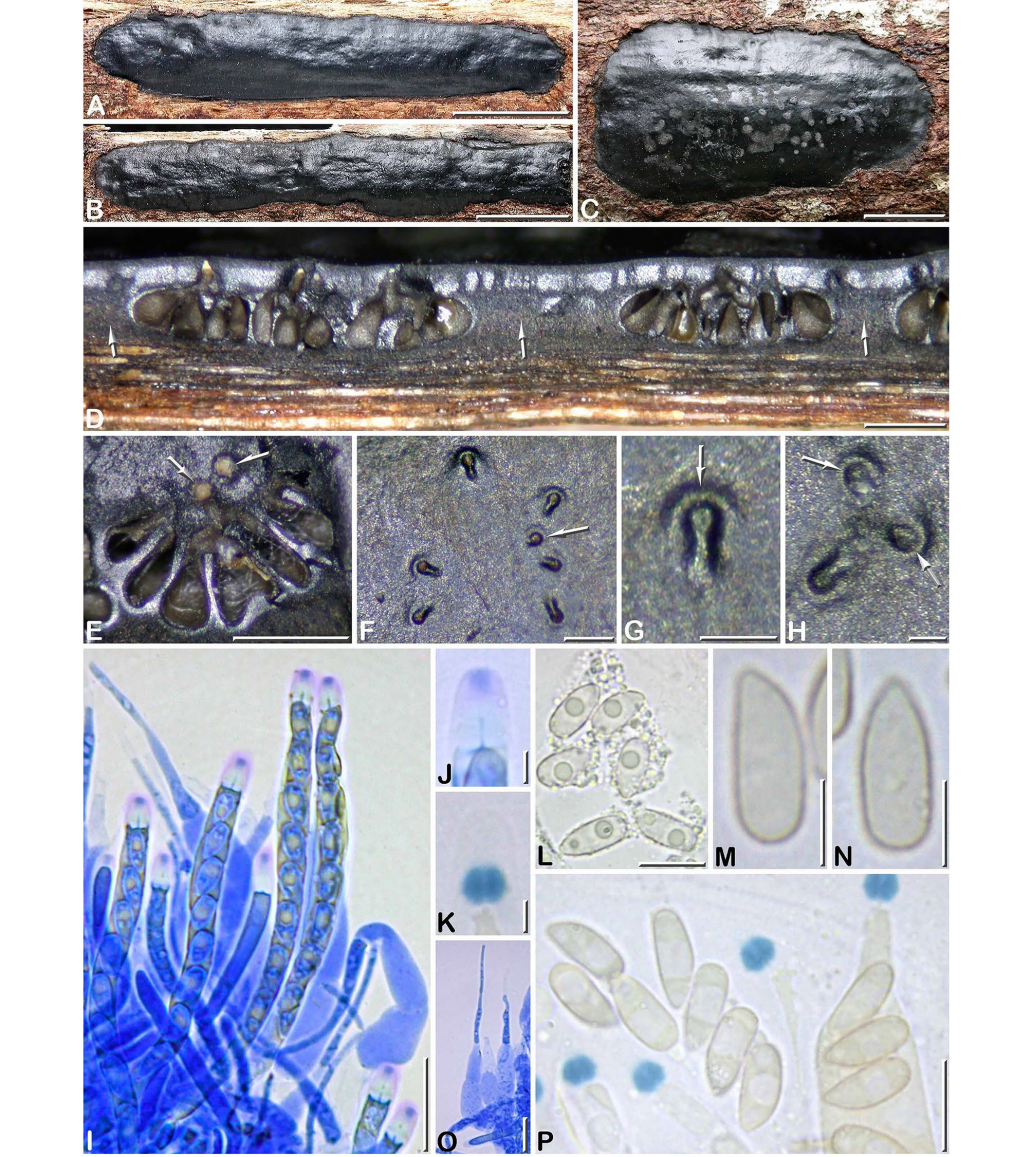
*Camillea nitida*. (A–D, I–P from holotype; E–H from paratype GYJF 12119). **A, B** Habit of two narrowly elongate stromata on host surface, showing a shiny surface; **C** Ellipsoid stroma in top view showing a shiny surface mottled with grey deposits of ascospores around the ostioles; **D** Stroma in vertical section showing clusters of perithecia with convergent ostiolar necks, separated by regions composed of brownish black sterile tissue (arrows); **E** Carbonaceous crust removed from the stromatal surface to show a rosette of empty carbonaceous perithecial cells arranged around two ostiolar canals (arrows); **F, H** Keyhole-shaped and rounded ostioles (arrows) in an outwardly radiating pattern; **G** Keyhole-shaped ostiole showing a slightly prominent rim (arrow); **I** Immature and mature asci and paraphyses, in blue Pelikan ink; **J** Ascus apical apparatus showing an apical plug stained by blue Pelikan ink; **K** Diamond-shaped subapical apparatus stained by Melzer’s reagent; **L** Freshly released ascospores with remnants of oily globules attached; **M, N** Ascospores respectively in side and dorsal view, in Melzer’s reagent; **O** Paraphyses in blue Pelikan ink; **P** Ascospores and apical apparati, in Melzer’s reagent. Scale bars: A, B = 10 mm; C = 5 mm; D, E = 0.5 mm; F = 0.2 mm; G, H = 0.1 mm; I, O = 20 μm; J, K = 2 μm; L, P = 10 μm; M, N = 5 μm

***Camillea nitida*** J. Fourn. & Y.-M. Ju sp. nov. Figs. 3C, 6

#### MycoBank MB 848866

##### Typification

FRENCH GUIANA: Maripasoula, Saül, shortcut to the airfield, 3.622159 N, 53.204166 W, disturbed mesophilic rainforest, ca. 210 m, on a dead corticated branchlet, 20 Jun. 2019, *Fournier, J.* GYJF 19123 (HAST 145961 holotype), GenBank: ITS = OQ871492, LSU = OQ871467.

##### Etymology

From Latin *nitidus* = shiny, for the conspicuously shining stromatal surface.

##### Diagnosis

Differs from *C. punctidisca*, morphologically the most resembling species, by narrowly elongate stromata 0.5–0.7 mm thick vs. orbicular with a raised margin and 1–2 mm thick, and keyhole-shaped ostioles vs. rounded.

Stromata erumpent through bark, (20–)40–100 mm long × 6–9(–12) mm wide, (0.4–)0.5–0.7 mm thick, applanate, slightly convex, narrowly elongate, occasionally irregularly ellipsoid, with a narrow, dull black, slightly sloping sterile margin; surface shiny black, even to slightly undulate with shallow depressions in places, minutely roughened by ostiolar rims; subsurface crust 200–250 μm thick, strongly carbonaceous, of alternating shiny black and dull black vertical bands; interperithecial tissue carbonaceous, black, and sterile tissue between groups of perithecia greyish brown to blackish, fibrous, soft-textured; subperithecial tissue lacking or reduced to a thin black layer; underlying bark tissues irregularly blackened in places. Perithecia subglobose to flask-shaped, laterally and basally flattened, 0.25–0.35 mm high × 0.20–0.25 mm diam., arranged in rosettes with ostiolar necks anastomosing under the crust and opening into a common ostiole. Ostioles randomly distributed or in sparse groups of 3–7 in an outwardly radiating pattern, slightly lower than surface, rounded ca. 60 μm diam. and surrounded by a low circular rim, to most often stretched out to become keyhole-shaped 100–125 μm long, with a low horse-shoe shaped rim.

Paraphyses hyphal, thin-walled, slightly longer than asci, swollen and septate at base, 9.0–13.0(–17.0) µm wide, abruptly narrowed into filiform apices tapering to 1.5–2.0 μm wide; perithecial content colorless. Asci narrowly cylindrical to slightly fusiform at maturity, with eight uniseriately arranged, slightly overlapping ascospores, the spore-bearing parts **(**60–)70–80(–90) × 6.0–7.0(–10.0) µm, subsessile with a sharp lower end attached to a small, rounded hymenial cell, extending to 25 μm in length, with conspicuous oily content filling the ascus between ascospores and fragmented into small oily guttules when ascospores are released; apical apparatus 6.5–8.5 μm high, bipartite, comprised of a rhomboid subapical apparatus 3.0–3.6 × 3.6–3.9 μm (Me = 3.3 × 3.8 μm, N = 25) with obtusely rounded angles, bluing in Melzer’s reagent and an inamyloid upper part with a short-cylindrical apical pulvillus 1.7 × 1.5 μm stained blue with a wide rosy halo by blue Pelikan ink.

Ascospores (8.5–)9.2–11.0(–12.3) × (3.4–)3.8–4.5(–4.7) µm, Q = (2.0–)2.1–2.9(–3.2), N = 120 (Me = 10.0 × 4.1 μm, Qe = 2.4), in side view rectangular with a beveled, narrowly rounded to subacute end and the other end broadly rounded, bullet-shaped in dorsal or ventral view, light yellowish, consistently oriented with the beveled end toward the base of the ascus, with a single oily droplet, rarely with 1–2 smaller ones; no germ slit visible; epispore smooth by LM, elongatedly reticulate-poroid by SEM (Fig. 3C).

##### Cultures and anamorph

Unknown.

##### Additional specimen examined (paratype)

FRENCH GUIANA: Régina, Nouragues natural reserve, Inselberg field centre, trail to Pararé 0.5 km from the camp, 4.311240 N, 52.132789 W, hygrophilic rainforest, 320–350 m asl, on a corticated branchlet, 21 Jun. 2012, *Fournier, J.* GYJF 12119 (HAST 145962) (partly depauperate).

##### Known distribution

French Guiana, known only from two collections.

**Notes.** *Camillea nitida* is distinct in having elongate, applanate stromata with a shiny surface and minute, scattered, low keyhole-shaped to horse-shoe shaped ostiolar rims. Further diagnostic features that make a unique combination supporting the status of *C. nitida* as a distinct species include: small perithecia arranged in rosettes with convergent and anastomosed ostiolar necks, short paraphyses with a swollen base and a subulate apex, apical apparatus with a large upper hyaline part, and rectangular ascospores with a beveled end, 10.0 × 4.1 μm on average.

Among *Camillea* species sharing with *C. nitida* stromata having a shiny surface with punctate ostioles and ascospores similar in shape and dimensions, *C. punctidisca*, known from Venezuela (Rogers 1980) (as *Hypoxylon punctidiscum*), is the most resembling species. However, the stromata of *C. punctidisca* differ by being orbicular with an abrupt raised margin that are thicker, 1–2 mm thick, and the ostioles are simply punctate, lacking the distinctive keyhole-shaped rim as encountered in *C. nitida.* Data on perithecial arrangement and ascus apex morphology were unfortunately not documented in the original description of *C. punctidisca.*

*Camillea punctulata* can likewise be considered for comparison, differing by a temperate North American distribution on *Quercus*. Moreover, it differs in featuring perithecia opening individually through minutely punctate ostioles that are widely scattered on stroma surface and smaller ascospores 7.0–9.0 × 3–4 μm (Læssøe et al. 1989)(Table 5).

**Table 5:** Ascospore dimensions from two collections of *C. nitida* compared with those of *C. punctidisca* and *C. punctulata* from literature

collection numbers	Ascospore measurements with extreme values in parentheses	Q = quotient l/w, N = number of measurements	Mean values
GYJF 12119 (paratype of *C. nitida*)	(8.5–)9.2–11.0(–12.3) × (3.6–)3.8–4.3(–4.5) µm	Q = (2.0–)2.2–2.9(–3.2), N = 60	Me = 10.0 × 4.1 μm, Qe = 2.4
GYJF 21123 (holotype of *C. nitida*)	(8.5–)9.2–10.8(–12.1) × (3.4–)3.8–4.5(–4.7) µm	Q = (2.0–)2.1–2.7(–3.1), N = 60	Me = 10.0 × 4.1 μm, Qe = 2.4
cumulated values	(8.5–)9.2–11.0(–12.3) × (3.4–)3.8–4.5(–4.7) µm	Q = (2.0–)2.1–2.9(–3.2), N = 120	Me = 10.0 × 4.1 μm, Qe = 2.4
* C. punctidisca* (Rogers 1980)	9.5–12 × 4.5–6 μm	–	Me = 10.8 × 5.3 μm, Qe = 2
* C. punctulata* (Læssøe et al. 1989)	7–9 × 3–4 μm	–	Me = 8.0 × 3.5 μm, Qe = 2.3

**Fig. 7:**
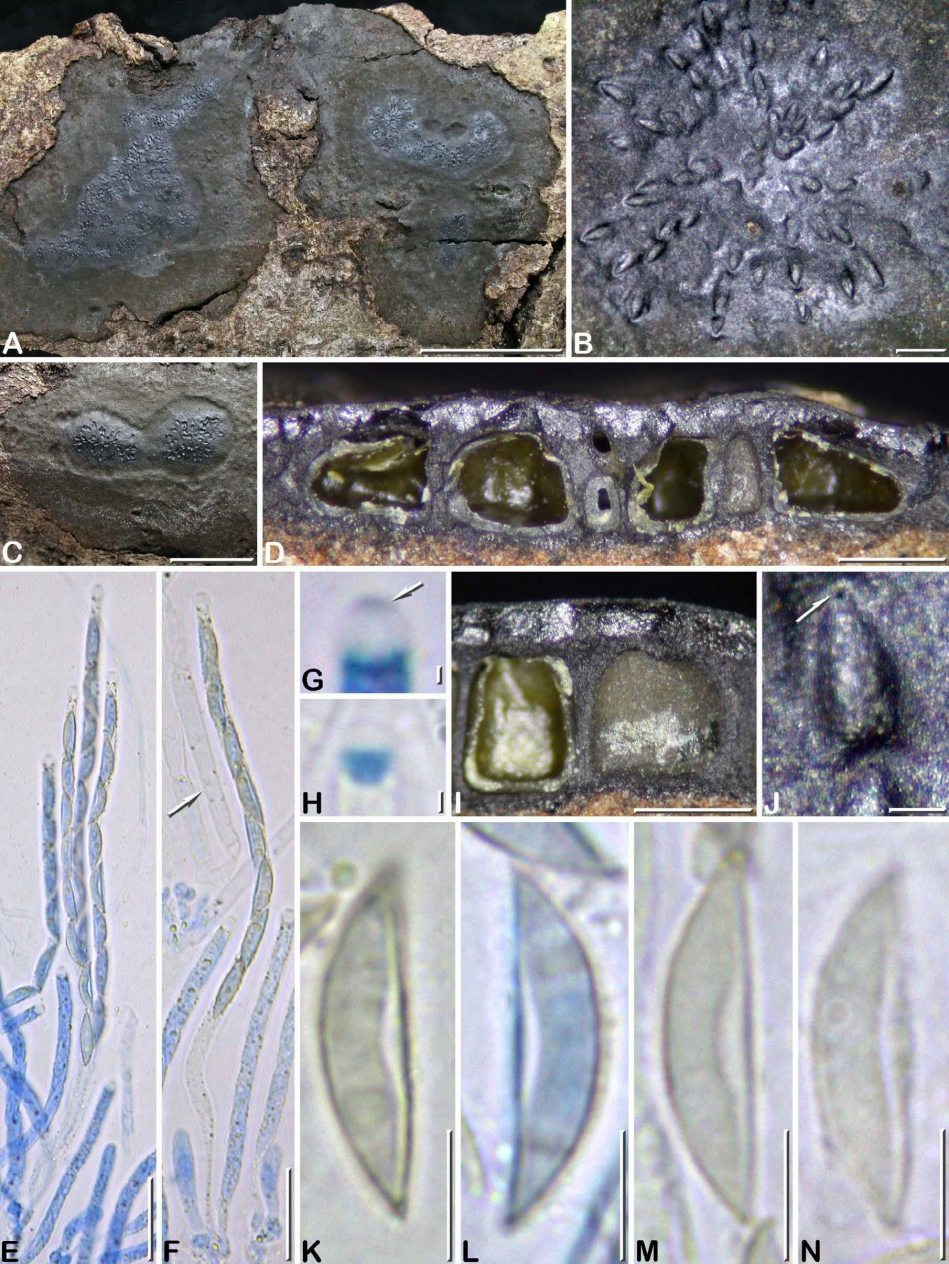
*Camillea rogersii* (from holotype). **A, C** Habit of stromata on host surface with wide sterile margins; **B** Close-up on the fertile part of a stroma showing an outwardly radiating arrangement pattern of ostiolar depressions; **D, I** Stroma in vertical section showing variously shaped perithecia with pale olivaceous content; **E, F** Asci in blue Pelikan ink, with paraphyses (arrow); **G** Ascus apex in blue Pelikan ink, showing a darker apical pulvillus (arrow); **H** Ascus apex showing the subapical apparatus bluing in Melzer’ reagent; **J** Ostiolar depression in close-up showing a minute apical ostiole (arrow) surrounded by a low rim; **K** Ascospore in side view in 1% SDS; **L** Ascospore in side view in Waterman blue ink diluted in lactic acid; **M, N** Ascospores in side view in Melzer’s reagent. Scale bars: A = 10 mm; B, D, I = 0.5 mm; C = 5 mm; E, F = 20 μm; G, H = 1 μm; J = 100 μm; K-N = 5 μm.

***Camillea rogersii*** J. Fourn. & Y.-M. Ju sp. nov. Figs. 3D, E, 5D–F, 7.

#### MycoBank MB 848867

##### Typification

FRENCH GUIANA: Maripasoula, Saül, trail head to Sentier des Gros Arbres, 3.6201 N, 53.207989 W, disturbed mesophilic rainforest, ca. 210 m, on a dead corticated branch, associated with depauperate stromata of *C. sulcata*, 27 Mar. 2021, *Fournier, J.* GYJF 21172 (HAST 145963 holotype), ex-type culture BRFM 3481, GenBank: ITS = OP595158, LSU = OP729385.

##### Etymology

In honor of the late Prof. Jack D. Rogers, for his invaluable contribution to the taxonomy of the Xylariales and his friendly encouragement of our work.

##### Diagnosis

Differs primarily from *C. amazonica* with which it shares similar minutely ellipsoid ostiolar depressions by inequilateral acute-fusiform ascospores 14.2 × 3.5 μm on average with a conspicuous ventral white line vs. ellipsoid, 11.4 × 5.3 μm and lacking a ventral white line.

Stromata erumpent through bark, applanate with widely spread, sterile, slightly sloping margins, orbicular to ellipsoid 10–15 mm diam. to irregular in outline by coalescence of several stromata, up to ca. 40 × 20 mm, 0.65–0.85 mm thick; fertile part central, orbicular to irregularly elongate, slightly convex, black, slightly shiny, contrasting with the wide, bronze-colored sterile margins; surface roughened by minute ellipsoid to bullet-shaped ostiolar depressions 0.25–0.60 mm long × 0.12–0.30 mm wide, radiating outward often in linear rows, with outward end curving upward and slightly raising stromatal surface to form a low rim, pierced by a minute ostiole 8–10 μm diam; subsurface crust 120–250 μm thick, strongly carbonaceous; interperithecial tissue dark brown to blackish, brittle. Perithecia subglobose to cuboid, basally and laterally flattened, asymmetrical at periphery, 0.40–0.60 mm high × 0.35–0.60 mm diam, opening individually through central or eccentric ostiolar necks, seated on a thin black carbonaceous layer.

Paraphyses hyphal, thin-walled, remotely septate, sparsely and minutely guttulate, 5.0–6.0 μm wide at base, tapering to 1.0–1.5 μm wide above asci; perithecial content pale olivaceous. Asci narrowly cylindrical, with 6–8 uniseriately arranged, slightly overlapping ascospores, the spore-bearing parts 90–100 × 3.8–4.5 μm, the stipes (18–)45–60 μm long, with slightly granular content, with a bipartite apical apparatus 2.5–3.5 μm high comprised of a trapezoid subapical apparatus attenuated at base, 1.1–1.5 × 2–2.3 μm (Me = 1.3 × 2.1 μm, N = 25), bluing in Melzer’s reagent and an inamyloid upper part with a small, discoid to lenticular apical pulvillus stained greyish blue by blue Pelikan ink.

Ascospores (12.7–)13.3–15.0(–16.5) × (3.0–)3.2–3.7(–3.9) µm, Q = (3.7–)3.8–4.5(–4.8), N = 60 (Me = 14.2 × 3.5 μm, Qe = 4.1), fusiform strongly inequilateral with acute ends, yellowish-grey, ventrally flat with a conspicuous white line almost spore-length, most often enlarged in median part; epispore smooth by LM, finely pitted by SEM (Fig. 3D, E).

##### Cultures and anamorph

Colonies 20 mm diam at 1 week, mycelium white, loosely cottony, medium stained brown under the colony, light olivaceous around the colony; 40 mm diam at 3 weeks, slightly pulvinate with fimbriate margins; mycelium white, cottony, superficially light brown in places at margins with age; reverse cinnamon (62); medium stained blackish brown; odor faint, sweetish. No conidiogenesis observed after 6 weeks.

##### Known distribution

French Guiana, known only from the type collection.

**Notes.** *Camillea rogersii* is diagnosed by the unique combination of applanate stromata with a wide olivaceous brown sterile margin, minute ellipsoid ostiolar depressions oriented outwardly, perithecia opening individually and with olivaceous content, small trapezoid subapical apparatus and acute-fusiform, strongly inequilateral light-colored ascospores 13.3–15.0 × 3.2–3.7 μm, smooth-walled by LM, finely pitted by SEM, featuring on the ventral side a long, conspicuous white line often enlarged in median part. The wall ornamentation observed by SEM is distinctive in showing extremely reduced isodiametric pores with thick septa. The SEM images do not explain the paler ventral line observed by LM by the presence of a particular structure, which suggests that it may result from the presence of an appressed sheath splitting on the ventral side of ascospores.

The most resembling species in outward appearance is *C. amazonica* Læssøe, J. D. Rogers & Whalley, which is primarily distinguished by smaller oblong-ellipsoid ascospores 10.3–12.2 × 4.7–5.6 μm, lacking a ventral white band (Læssøe et al. 1989). The epispore ornamentation was shown by these authors to be reticulate-poroid, while that of *C. rogersii* is finely pitted.

*Camillea heterostomoides* described herein displays a similar stromatal surface with small, shallow ellipsoid ostiolar rims like *C. rogersii* but mainly differs by larger, fusiform, slightly inequilateral and heteropolar ascospores 21.2–26.3 × 6.5–7.4 μm. Several species around *C. stellata* and *C. labellum*, including *C. saulensis* described below and *C. venezuelensis* (J. H. Miller) Dennis, share certain common features, i.e., acutely fusiform-inequilateral ascospores frequently featuring a ventral, paler linear zone (JF pers. observations); these species can be distinguished by their stromatal shape, ascospore dimensions, and ostiolar morphology. *Camillea rogersii* shares with *C. stellata* ostioles located in ellipsoid ostiolar depressions and ascospores in the same size range (Læssøe et al. 1989) but differs in having applanate stromata and small cuboid perithecia. Our ITS-LSU based phylogram (Fig. 1) suggests that they are distantly related, with *C. rogersii* being shown to have a closer affinity with *C. labellum*, which differs by short-cylindrical stromata with a prominent upper rim and minutely umbilicate ostioles. *Camillea saulensis* can be distinguished from *C*. *rogersii* by shiny black stromata with conic-papillate ostioles, and *C. venezuelensis* by thick stromata with vertical sides, a prominent upper rim, and larger ascospores (Læssøe et al. 1989).

**Fig. 8:**
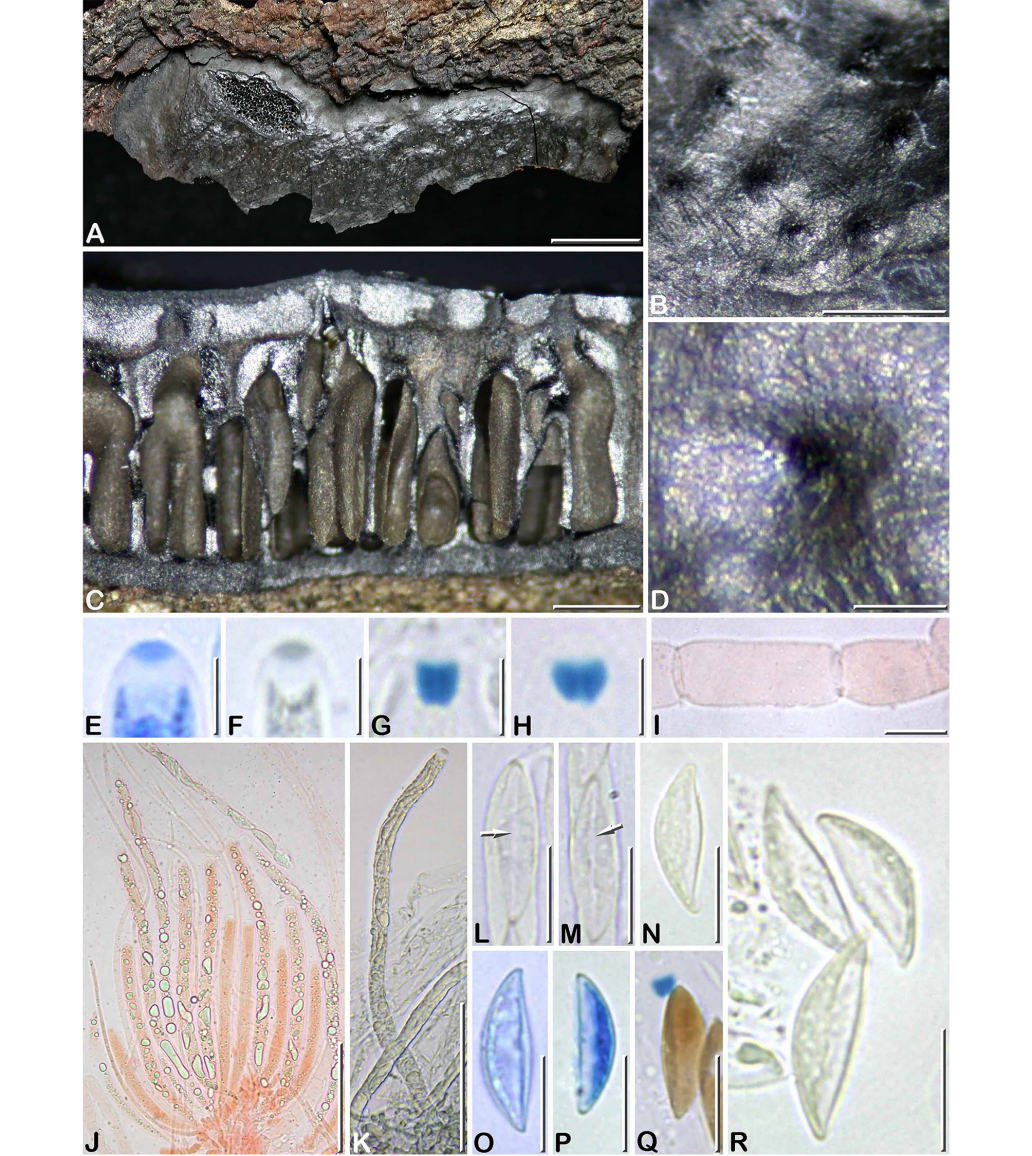
*Camillea saulensis* (from holotype). **A** Habit of a fragmentary stroma erumpent from bark; **B** Close-up on the stromatal surface showing scattered papillate ostioles; **C** Stroma in vertical section showing tubular to flask-shaped perithecia sharing common ostioles; **D** Close-up on a papillate ostiole; **E** Ascus apex in blue Pelikan ink, showing an apical pulvillus stained blue; **F** Ascus apex in blue black Waterman ink, showing an apical pulvillus stained dark grey; **G, H** Ascus apices showing the subapical apparatus bluing in Melzer’ reagent; **I** Basal part of a paraphysis, in Congo red; **J** Bundle of immature and mature asci showing refractive content, in Congo red; **K** Mature ascus in blue black Waterman ink; **L, M** Two barely mature ascospore in ventral view showing a narrow straight white line (arrows), in Melzer’s reagent; **N** Ascospore in side view, in 1% SDS; **O, P** Ascospores in side view showing a ventral paler area of variable width, in blue Pelikan ink; **Q** Atypical ascospores with pigmented wall, in Melzer’s reagent; **R** Discharged ascospores showing a wide, unpigmented ventral zone, in 1% SDS. Scale bars: A = 10 mm; B, C = 0.5 mm; D = 0.2 mm; E-H = 5 μm; I, L-R = 10 μm; J, K = 50 μm

***Camillea saulensis*** J. Fourn. & Y.-M. Ju sp. nov. Figs. 3F, G, 8

#### MycoBank MB 848868

##### Typification

FRENCH GUIANA: Maripasoula, Saül, trail head to Roche-Bateau, 3.620498 N, 53.199309 W, disturbed mesophilic rainforest, ca. 240 m, on a recently dead corticated trunk, 22 Jun. 2019, *Fournier, J.* GYJF 19203 (HAST 145964 holotype), GenBank: ITS = OQ871493, LSU = OQ871468.

##### Etymology

For Saül, the holotype location.

##### Diagnosis

Differs from other known species of *Camillea* with large and thick applanate stromata by the combination of an uneven, shiny black surface with finely papillate ostioles, cylindrical intricate perithecia sharing common ostioles and light-colored, inequilateral-fusiform ascospores 15.5 × 4.2 μm on average, with acute ends and a white ventral zone becoming wider with age.

Stroma fragmentary, erumpent through bark, applano-pulvinate, irregularly ellipsoid, 55 × 27 mm, including a sterile sloping margin 3–7 mm wide, up to 1.5–1.7 mm thick at the center; fertile part central, shiny black, contrasting with the dull grey sterile margin; surface uneven with shallow irregular depressions, finely roughened by scattered ostioles; subsurface crust 130–290 μm thick, strongly carbonaceous; interperithecial tissue entirely carbonaceous, black, connected to a basal carbonaceous layer 120–170 μm thick, seated on inner bark tissue. Perithecia narrowly cylindrical to flask-shaped, 0.8–1 mm high × 0.20–0.35 mm diam, somewhat intricate, anastomosing by 2–3 into a wide common ostiolar canal. Ostioles widely scattered, finely papillate, obtusely rounded to conical with an apical pore ca. 80 μm diam.

Paraphyses copious, hyphal, thin-walled, septate, sparsely and minutely guttulate, 9.0–11.0 μm wide at base, tapering to 1.0–1.5 μm wide above asci; perithecial content colorless. Asci narrowly cylindrical, short-stipitate, with 6–8 uniseriately arranged, slightly overlapping ascospores, 120–160 μm in total length × 5.0–5.5 μm, the stipes 15–20 μm long, with oily guttules coalescing into large refractive bodies separating ascospores; with a bipartite apical apparatus 3.5–5.0 μm high, comprised of a more or less broadly trapezoid subapical apparatus attenuated at base, 2.2–2.8 × 2.4–2.9 μm (Me = 2.5 × 2.6 μm, N = 25), bluing in Melzer’s reagent, and an inamyloid upper part with a lenticular apical pulvillus 1.1–1.5 × 2.5–3 μm (Me = 1.3 × 2.7 μm, N = 15) stained blue by blue Pelikan ink or dark grey in blue-black Waterman ink, unstained by Congo red (Fig. 8E, F).

Ascospores (13.5–)14.2–16.5(–18) × (3.2–)3.8–4.8(–5) µm Q = (2.9–)3.3–4 (–5.1), N = 60 (Me = 15.5 × 4.2 μm, Qe = 3.7), fusiform strongly inequilateral with acute ends, light yellowish-grey, occasionally brown, ventrally flat or slightly convex with a narrow white line spore-length, evolving to a wide unpigmented ventral area becoming conspicuous with age; epispore smooth to finely roughened by LM, minutely reticulate-poroid with isodiametric to slightly elongated pores by SEM (Fig. 3F, G).

##### Cultures and anamorph

Unknown.

##### Known distribution

French Guiana, known only from the type collection.

**Notes.** *Camillea saulensis* displays the unique combination of thick applano-pulvinate stromata with a shiny black surface roughened by minutely papillate ostioles, narrowly cylindrical perithecia anastomosed beneath the surface, small trapezoid subapical apparatus and acute-fusiform, strongly inequilateral light-colored ascospores 14.2–16.5 × 3.8–4.8 μm, smooth-walled by LM, finely reticulate-poroid by SEM, featuring on the ventral side a narrow white zone gradually spreading on the sides with age.

As discussed under *C. rogersii* herein, a similar ascospore morphology is encountered in species related to *C. labellum* such as *C. stellata* and *C. venezuelensis*, with the unusual presence of variously shaped white ventral zones in *C. rogersii*, *C. labellum*, and *C. stellata* and presence of a short ventral thickening in *C. venezuelensis* (JF, unpublished data). *Camillea saulensis* is readily set apart from these four species by the combination of stromatal, ostiolar and perithecial characters, and by the finely reticulate-poroid epispore by SEM, which are longitudinal twisted ribbed in these four species. *Camillea hainesii* is the only species featuring, like *C. saulensis*, stromata with a shiny surface and papillate ostioles. It differs by smaller, rectangular ascospores with lower end beveled, 8.3 × 4.1 μm on average, asci with a rhomboid subapical apparatus and smaller ostioles opening individually (Læssøe et al. 1989; Rogers and Dumont 1979).

## Conclusion

The genus *Camillea* was first established by Fries (1849), based on eight species described as *Hypoxylon* by Montagne (1840) from French Guiana. Despite the limited duration and sampling area of the current study in French Guiana, five new species were discovered and compared in Table 6. This signifies that there is a substantial *Camillea* diversity yet to be explored in French Guiana and adjacent neotropical forests. The discovery of these new species highlights the importance of continued research and exploration in these regions to better understand and document the *Camillea* biodiversity present.

**Table 6:** Synopsis of morphological characters differentiating the five new *Camillea* species

	*C. cribellum*	*C. heterostomoides*	*C. nitida*	*C. rogersii*	*C. saulensis*
**Surface**	Dull black	Dull black	Shiny black	Dull black with a bronze margin	Shiny black
**Thickness**	0.45–0.5 mm	0.4–0.5 mm	0.5–0.7 mm	0.5–0.85 mm	1.5–1.7 mm
**Ostioles**	Minutely umbilicate	In wedge-shaped depressions	In keyhole-shaped rims	In narrowly ellipsoid depressions	Papillate
**Perithecial arrangement**	Opening individually	Opening individually	Opening collectively	Opening individually	Opening collectively
**paraphyses**	Refractive guttules	Minutely guttulate	Swollen at base	Minutely guttulate	Minutely guttulate
**Spore dimensions and colour**	7–9 × 3.5–4.4 μm, light yellowish	21.2–26.3 × 6.5–7.4 μm, yellowish	9.2–11 × 3.8–4.5 μm, light yellowish	13.3–15.0 × 3.2–3.7 μm, yellowish grey	14.2–16.5 × 3.8–4.8 μm, light yellowish grey
**Spore shape**	Rectangular with one attenuated end	Fusiform heteropolar	Rectangular with one attenuated end	Fusiform-inequilateral with acute ends	Fusiform-inequilateral with acute ends
**Ornamentation by SEM**	Angular reticulate-poroid	Reticulate-poroid	Elongatedly reticulate-poroid	Finely pitted	Minutely reticulate-poroid

## Data Availability

Specimens are deposited at the herbarium HAST. Cultures are available at BRFM. DNA sequences are deposited at GenBank.

## References

[CR1] Dennis RWG (1957). Further notes on tropical american Xylariaceae. Kew Bull.

[CR201] Dennis RWG (1970) Fungus flora of Venezuela and adjacent countries. Kew Bulletin Additional Series 3:344–531

[CR2] Fournier J (2022). *Camillea lechatii* (Graphostromataceae, Xylariales), a new species from Martinique (French West Indies). Ascomycete org.

[CR3] Fries EM (1849) Summa vegetabilium Scandinaviae, sectio posterior. Holmiae & Lipsiae, pp 261–572

[CR4] Hastrup ACS, Læssøe T (2009). *Camillea* (Xylariaceae, Ascomycota), including two new species, along a trans-andean altitude gradient in Ecuador. Mycological Progress.

[CR5] Hsieh H-M, Ju Y-M, Hsueh P-R, Lin H-Y, Hu F-R, Chen W-L (2009). Fungal keratitis caused by a new filamentous hyphomycete *Sagenomella keratitidis*. Bot Stud.

[CR202] Hsieh H-M, Lin C-R, Fang M-J, Rogers JD, Fournier J, Lechat C, Ju Y-M (2010) Phylogenetic status of *Xylaria* subgen. *Pseudoxylaria* among taxa of the subfamily Xylarioideae (Xylariaceae) and phylogeny of the taxa involved in the subfamily. Molecular Phylogenetics and Evolution 54:957–969 10.1016/j.ympev.2009.12.015 20035889

[CR8] Læssøe T, Rogers JD, Whalley AJS (1989). *Camillea*, *Jongiella* and light-spored species of *Hypoxylon*. Mycol Res.

[CR7] Li QR, Gong XF, Zhang X, Pi YH, Long SH, Wu YP, Shen XC, Kang YQ, Kang JC (2021). Phylogeny of Graphostromatacea with two new species (*Biscogniauxia glaucae* sp. nov. and *graphostroma guizhouensis* sp. nov.) and new record of *Camillea broomeana* isolated in China. Arch Microbiol.

[CR203] Lloyd CG (1918) Mycological notes no. 54. Mycological Writings 5:765–780

[CR9] Miller JH (1961). A monograph of the world species of *Hypoxylon*.

[CR10] Montagne JPFC (1840). Seconde centurie de plantes cellulaires exotiques nouvelles, décades III, IV et V. Annales des sciences naturelles. Botanique Sér II.

[CR11] Montagne JPFC (1855) Cryptogamia Guyanensis, seu Plantarum cellularium in Guyana gallica annis 1835–1849 a Cl. Leprieur collectarum enumeratio universalis. Annales des sciences naturelles, Botanique, Sér IV, 3:91–144 + plates 5 & 6

[CR12] Nylander JAA (2004) MrModeltest v2. Program distributed by the author. Evolutionary Biology Centre, Uppsala University

[CR13] Pouzar Z (1979). Notes on taxonomy and nomenclature of *Nummularia* (Pyrenomycetes). Česká Mykologie.

[CR14] Rogers JD (1968). Nuclear phenomena in the ascospores of *Hypoxylon punctulatum*. Can J Bot.

[CR15] Rogers JD (1975). The ascospore of *Hypoxylon glycyrrhim*. Mycologia.

[CR16] Rogers JD (1975). *Nummularia broomeiana*: conidial state and taxonomic aspects. Am J Bot.

[CR17] Rogers JD (1977). Surface features of the light-colored ascospores of some applanate *Hypoxylon* species. Can J Bot.

[CR19] Rogers JD (1980). On the types of three putative species of *Eutypa* described by George Massee. Mycologia.

[CR18] Rogers JD, Dumont KP (1979). Los Hongos de Colombia VI. Two new applanate species of *Hypoxylon*. Mycologia.

[CR20] Rogers JD, Læssøe T, Lodge DJ (1991). *Camillea*: new combinations and a new species. Mycologia.

[CR21] Rogers JD, San Martín F, Ju Y-M, Hansen K (2000). Venezuelan fungi: *Biscogniauxia viscosicentra* sp. nov. and the anamorph of *Camillea cyclops*. Nova Hedwigia.

[CR22] Rogers JD, San Martín F, Ju Y-M (2002). Three new taxa of *Camillea* from Costa Rica. Sydowia.

[CR23] Rogers JD, Vasilyeva L, Hay FO (2008). New Xylariaceae from Hawaii and Texas (USA). Sydowia.

[CR24] Ronquist F, Teslenko M, van der Mark P, Ayres DL, Darling A, Hohna S, Larget B, Liu L, Suchard MA, Huelsenbeck JP (2012). MrBayes 3.2: efficient bayesian phylogenetic inference and model choice across a large model space. Syst Biol.

[CR25] San Martín F, Rogers JD (1993). *Biscogniauxia* and *Camillea* in Mexico. Mycotaxon.

[CR205] Spegazzini C (1889) Fungi Puiggariani. Boletin de la Academia Nacional de Ciencias en Córdoba, República Argentina 11:381–622

[CR26] Stamatakis A (2014). RAxML version 8: a tool for phylogenetic analysis and post-analysis of large phylogenies. Bioinformatics.

[CR27] Thompson JD, Gibson TJ, Plewniak F, Jeanmougin F, Higgins DG (1997). The CLUSTAL X windows interface: flexible strategies for multiple sequence alignment aided by quality analysis tools. Nucleic Acids Res.

[CR206] Vasilyeva LN, Stephenson SL, Hyde KD, Bahkali AH (2012) Some stromatic pyrenomycetous fungi from northern Thailand-1. *Biscogniuxia, Camillea,* and *Hypoxylon* (Xylariaceae). Fungal Diversity 55:65–76

[CR28] Vilgalys R, Hester M (1990). Rapid genetic identification and mapping of enzymatically amplified ribosomal DNA from several *Cryptococcus* species. J Bacteriol.

[CR29] Wendt L, Sir EB, Kuhnert E, Heitkamper S, Lambert C, Hladki AI, Romero AI, Luangsa-ard JJ, Srikitikulchai P, Persoh D (2018). Resurrection and emendation of the Hypoxylaceae, recognised from a multigene phylogeny of the Xylariales. Mycological Progress.

[CR207] Whalley MA (1995) *Camillea fusiformis* sp. nov. from Ecuador. Sydowia 47:82–88

[CR208] Whalley MA, Whalley AJS, Jones EBG (1996) *Camillea selangorensis* sp. nov. from Malaysia. Sydowia 48:145–151

[CR209] Whalley MA, Whalley AJS, Thienhirun S, Sihanonth P (1999) *Camillea malaysianensis* sp. nov. and the distribution of *Camillea* in Southeast Asia. Kew Bulletin 54:715–722

